# Hepatitis C Virus and Lichen Planus: The Real Association

**Published:** 2010-09-01

**Authors:** Nima Mahboobi, Farzaneh Agha-Hosseini, Kamran Bagheri Lankarani

**Affiliations:** 1 Department of Oral and Maxillofacial Surgery, School of Dentistry, Tehran University of Medical Sciences, Tehran, Iran; 2 Department of Oral Medicine, School of Dentistry, Tehran University of Medical Sciences, Tehran, Iran, aghahose@sina.tums.ac.ir; 3 Health Policy Research Center, Shiraz University of Medical Sciences, Shiraz, Iran, lankarani@mohme.gov.ir

Lichen planus (LP) is a common T-cell-mediated chronic inflammatory disease of the stratified squamous epithelium, with unknown etiology. It can affect oral mucosa, the skin, genitalia, hair follicles, nails, esophagus, urinary tract, nasal mucosa, larynx and even the eyes [1]. Local conditions such as poor oral hygiene and smoking may increase the chance of the immune trigger by increasing the exposure. Oral LP (OLP) affects women more than men [1][2] and occurs predominantly in adulthood, although young people and children might be affected [1]. Clinically, the OLP has six variants: Papular, reticular, plaque-like, atrophic, erosive and bullous. These features may occur individually or in combination [2]. There is ongoing concern that OLP may be premalignant [3]. While skin lesions occur in 20% of patients with OLP, cutaneous lesions are associated with oral lesions in 70%–77% of cases [4]. The oral mucosa in OLP is highly accessible for an accurate examination. Therefore, OLP is ideal for the study of human  -cell-mediated inflammation and autoimmunity [5][6][7]. Oral lesions are characteristically raised multiform white lesions, accompanied by areas of erosions and pigmentation [1]. Histological features of the LP are nonspecific and there are no well-accepted criteria for its diagnosis [8] which makes its definite diagnosis difficult.Hepatitis C virus (HCV) is a single-stranded RNA virus which is recognized as a global concern [9]. Worldwide, more than 170 million people are infected with HCV [10]. The virus has an extremely variable genome, six distinct genotypes and multiple subtypes [5]. It is estimated that 0.16% of the Iranian general population are infected with the virus [11]. Infection with HCV has been found to be a major cause of liver diseases. Although the incidence of HCV infection is significantly lower than that of hepatitis B virus (HBV) infection, the rate of chronically infected individuals is much higher [12]. Morbidity associated with HCV infection is not only due to the sequelae of chronic liver disease, but is also due to a variety of extrahepatic manifestations [5]. There is no efficient vaccine available and it seems too optimistic to predict one in the near future. More epidemiologic studies are needed to better assess the epidemiological characteristic of the disease [13]. Correlation between HCV infection and some oral diseases such as OLP, Sjögren’s syndrome, and sialadenitis has been reported. Moreover, OLP was found associated with a number of viral infections including Epstein-Barr virus, cytomegalovirus, varicella zoster virus, human herpes virus , human papilloma virus, and human immunodeficiency virus (HIV). However, the most frequent evidence relates to HCV infection [14][15]. If this would be a true association, OLP in certain populations may be used as a sign of HCV infection in asymptomatic patients, leading to early diagnosis and treatment, and possibly a better prognosis of the infected patients [5][14][16]. The first description of this association was reported in 1991, just two years after discovery of HCV [17]. During the past years, studies from Taiwan [18], Brazil [19], Israel [20], Saudi Arabia [21], Turkey [22], Iran [23] and Thailand [24] showed statistically significant correlation between presence of LP and HCV infection. A study from Japan reported that the prevalence of OLP increased as the subjects grew older [25]. On the other hand, many researchers found no correlation between chronic HCV infection and LP. An Italian study showed just a weak relation between HCV infection and OLP [26]. Another two Italian studies [27][28], two Indian studies [29][30] two Iranian studies [31][32], one Brazilian study [33], one Turkish study [34], one Serbian study [35] and one from UK [36] were not able to find any correlation between chronic HCV infection and LP. A recent meta-analysis exploring the association between HCV and LP, nonetheless, revealed an important association. The pooled odds ratio point estimate of the prevalence of HCV infection among patients with LP was 5.4 (95% confidence interval [CI]: 3.5–8.3), compared to the control subjects. The odds ratio for LP among patients with HCV compared to control participants was 2.5 (95% CI: 2.0–3.1) [37]. The most likely hypothesis describing the association of HCV infection and LP is regionalbased correlation [37][38]. Nevertheless, it seems very superficial to just conclude a simple geographical correlation. This non-homogeneity in results from different geographic areas may have several reasons. Many of these reports for LP come from registries of hospitals or university affiliated clinics. These cannot represent the real situation in the general population for sure. Difficulties in making a definite diagnosis for LP—as previously mentioned—make interpretation even more complex. Estimation of the point prevalence of HCV infection in the general population in these regions and how well the control group was selected are other contributing variables which may lead to divergent results. Also, as HCV treatment, especially interferon-α, may provoke oral lesions similar to OLP [39], lack of information on the treatment status of enrollees with HCV infection in many of these studies makes summarizing the results challenging [37]. Analysis of available data revealed that it is too premature to reach a definite conclusion. So far, the most plausible path for this association is based on various factors such as region. Furthermore, we need to find the underlying mechanisms for the association. Experimental data strongly suggest that HCV is involved in the pathogenesis of OLP through local induction of an immune response specific for HCV epitopes [5]. HCV RNA has been detected both in sera and in oral lesions of patients with OLP; however no direct pathogenic effect of HCV on oral mucosa could be demonstrated [40][41][42]. Theoretically, epitopic similarities between HCV and keratinocytes could explain the association between LP and HCV, but this could not be demonstrated in any studies. It is believed that this association might be related to cytotoxic immune response to epithelia cells infected with HCV [41][42][43]. In some of these reports, HCV infected patients with LP had a higher serum transaminase level, and a higher chance of being diabetic than those without LP [37][44]. On the other hand, oral lesions in patients with HCV infection with LP were more likely to be erosive, when compared to non-infected LP patients [45][46][47]. This may reflect a synergistic effect between the two conditions. Interestingly, co-infection with HIV decreases the possibility  of LP in HCV-infected patients, probably through an immunodeficiency state [48][49]. It should be mentioned that no correlation was observed between the viral load and HCV genotype and the likelihood of developing LP in HCV-infected patients [50][51][52]. Overall, it can be concluded that HCV-infected patients may have increased risk of developing LP or alternatively, patients with LP may be at a higher risk for developing HCV infection [38]. Altogether, screening OLP patients for antibodies to HCV is recommended [20]. More prospective well-designed studies (especially cohorts) are necessary to clarify the above issue. There are some reports of association between LP and other chronic liver diseases including primary biliary cirrhosis, and cirrhosis of unknown origin. This may indicate other modes of interaction. Why is this association important? As HCV infection is usually indolent so that patients may present only in late stages of the disease with serious complications like cirrhosis and chronic liver disease,screening of patients with LP may help in early diagnosis of the HCV infection in a subset of patients. Early diagnosis, education and awareness of these patients may decrease risk of transmission to others. In a cost-effectiveness analysis, screening of patients with LP with ELISA was found cost-effective only with the presence of other risk factors such as history of intravenous drug abuse (IVDU), sex with IVDU , or history of transfusion [53][54].Reviewing these findings may help us in better understanding the pathogenesis of HCV infection, especially its extrahepatic manifestations. It should be emphasized that the epidemiologic studies could not prove any causative role for HCV in LP.
